# High Resolution Imaging of DNA Methylation Dynamics using a Zebrafish Reporter

**DOI:** 10.1038/s41598-017-05648-8

**Published:** 2017-07-14

**Authors:** Ranran Zhang, Lian Liu, Yuxiao Yao, Fei Fei, Feng Wang, Qian Yang, Yonghao Gui, Xu Wang

**Affiliations:** 1 Cardiovascular Center, Children’s Hospital of Fudan University, Shanghai, 201102 China; 2 Key Laboratory of Metabolism and Molecular Medicine, Ministry of Education, Department of Biochemistry and Molecular Biology, School of Basic Medical Sciences, Fudan University, Shanghai, 200032 China

## Abstract

As one of the major epigenetic modifications, DNA methylation is constantly regulated during embryonic development, cell lineage commitment, and pathological processes. To facilitate real-time observation of DNA methylation, we generated a transgenic zebrafish reporter of DNA methylation (zebraRDM) via knockin of an mCherry-fused methyl-CpG binding domain (MBD) probe driven by the *bactin2* promoter. The probe colocalized with heterochromatin, and its intensity was positively correlated with 5 mC immunostaining at a subcellular resolution in early embryos. Biochemical assays indicated that cells with stronger fluorescence maintained a higher level of DNA methylation, and time-lapse imaging at the blastula stage showed that the level of DNA methylation was transiently strengthened during mitosis. By crossing zebraRDM with other fluorescent transgenic lines, we demonstrate that the reporter can visually distinguish different cell lineages in organs like the heart. Our zebraRDM reporter therefore serves as a convenient and powerful tool for high-resolution investigation of methylation dynamics in live animals.

## Introduction

Epigenetics, including DNA methylation and histone modification, plays a pivotal role in regulating embryonic development. In vertebrates, methylation of CpG islands in promoter regions is important to regulate gene expression and maintain chromatin stability. Methylation patterns undergo dynamic changes during embryonic development and organogenesis, especially in genomically imprinted genes^1–3^. Aberrant DNA methylation is related to carcinogenesis and other pathological processes^4–6^. Methylated CpGs are recognized by methyl-CpG-binding proteins (MBPs), including MBD1, MBD2, MBD3, MBD4 and MeCP2^7^. The MBPs share a homologous conserved methyl-CpG-binding domain (MBD), and otherwise differ in their domain composition, containing domains such as zinc finger motifs and powerful transcriptional repressor domains (TRDs)^8–10^. The MBD domain mediates the capacity to bind single, symmetrically methylated CpG dinucleotides, and based on results from a functional binding mapping assay performed on all MBD family members, MBD1 bears the most sensitive MBD domain in response to changes in DNA methylation^7, 11^.

Several approaches have been developed to study DNA methylation. Traditionally, DNA methylation status is measured by detecting 5-methylcytosine (5 mC) or MBPs, through immunohistochemistry, methylated DNA immunoprecipitation (MeDIP), methylated-CpG island recovery assay (MIRA), and bisulfite sequencing^12–16^. However, these biochemical strategies only provide a snapshot of DNA methylation, which changes dynamically, and can only reflect the average condition of a cell population. Furthermore, these techniques require considerable processing, such as fixation, cryosection, acid treatment, antibody binding, etc., which is time-consuming and may introduce bias^13^.

Considering the vital impact of DNA methylation on pathogenesis, and the possibility for therapeutic exploration, an *in vivo* reporter system that facilitates the dynamic investigation of DNA methylation would be a valuable tool. Recently, efforts have been made to achieve real-time observation of DNA methylation *in vivo*. Several transgenic mouse reporters have been generated, including the MethylRO mouse, which uses a GFP/RFP-fused MBD probe, and the RGM mouse, which uses a methylation-sensitive reporter system based on a minimal imprinted promoter^17, 18^. Studies based on these reporters have illustrated DNA methylation dynamics in early embryonic cells and embryonic stem cells (ESCs) by direct monitoring of cellular behavior. Zebrafish share 70% of their genes with humans, and are more economical than small mammals. They are an ideal model organism for live imaging because of their optical clarity and rapid development, allowing the study of development and pathogenesis in a short time^19^.

In this study, we have generated the transgenic zebrafish line *Tg*(*bactin2:mCherry-MBD-IRES-nlsEGFP*), which expresses a fused probe mCherry-MBD under the control of a ubiquitous promoter^20^. Using this probe, we have achieved high-resolution imaging of heterochromatin structure, and verified the correlation of mCherry-MBD with 5 mC. By studying differential methylation status in various cell lineages, such as Wnt-responsive cells and differentiated cardiac cells, we observed that DNA methylation contributes to the differentiation of stem cells and the commitment of progenitor cells. This model promises to be a powerful visual tool, providing a deeper understanding of DNA methylation dynamics in stem cell and developmental biology.

## Results

### Generation of a viable and fertile transgenic zebrafish line ubiquitously expressing mCherry-MBD

**Figure 1:**
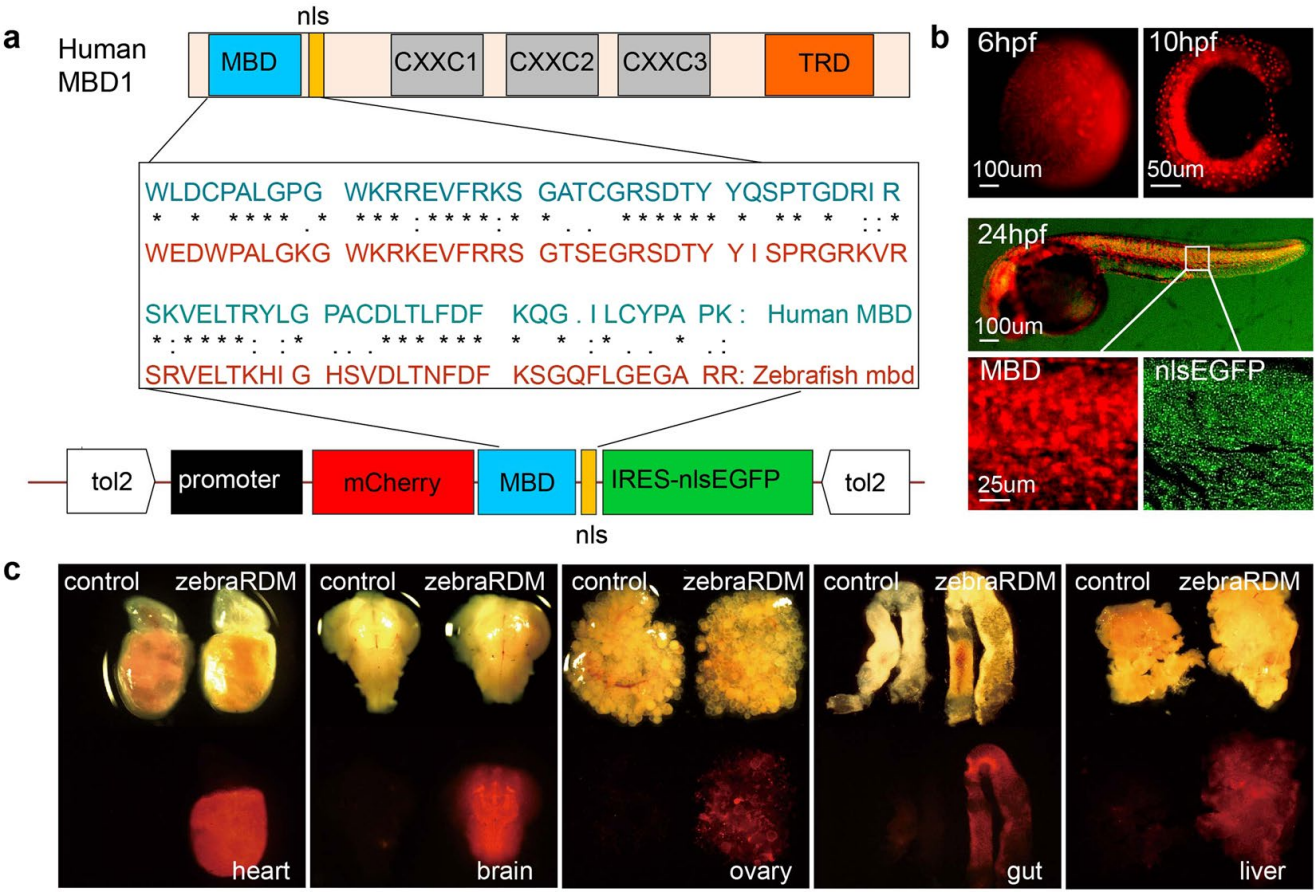
Generation of a zebrafish reporter ubiquitously expressing mCherry-MBD. (**a**) The structure of human MBD1, a comparison of the amino acid sequences of human and zebrafish methyl-binding domains, and the Tol2-based vector structure including promoter, the mCherry-MBD probe, and IRES-nlsEGFP. MBD, methyl binding domain; nls, nuclear localization signal; CXXC, cysteine-rich domains; TRD, transcriptional repression domain. (**b**) Fluorescence images of Tg(*bactin2:mCherry-MBD-IRES-nlsEGFP*) F1 embryos at 6, 10, and 24 hpf. Magnified images in white boxes display the mCherry-MBD channel (left) and IRES-nlsEGFP channel (right). (**c**) The distribution of mCherry-MBD in the adult zebrafish heart, brain, liver, ovary, and gut. The reporter is labeled as zebraRDM.

MBD1 is the largest MBD family member, containing one MBD domain, three zinc finger motifs (CXXC1, CXXC2, and CXXC3) and one TRD domain^8, 9, 21^. Previously, to visualize the DNA methylation status in mouse ESCs, the CXXX and TRD domains were removed to avoid potential binding to non-methylated regions, and the remaining MBD domain with endogenous nuclear localization sequence were fused to a fluorescent protein (mCherry/EGFP-MBD)^20, 22, 23^. MBD domains are highly conserved in evolution among vertebrates (Fig. 1a), and here the human MBD-based probe was used to generate a zebrafish reporter line. It was predicted that the probe may cause a dosage-dependent lethal effect, and therefore it was critical to identify a proper ubiquitous promoter to generate a stable transgenic line^17^. A total of three available ubiquitous promoters (*bactin2*, *ef1a* and *h2afx*) in zebrafish were selected to induce expression of the probe throughout the body, and the recombinant vectors were constructed via Tol2kit^24, 25^.

In detail, the mCherry-MBD was linked behind each of the three ubiquitous promoters, followed by an IRES-driven nlsEGFP (Fig. 1a), which served as both a nuclear label and a comparison for the distribution pattern of mCherry-MBD. The plasmid was co-injected with *tol2* transposase mRNA into zebrafish embryos at the single-cell stage. In the F0 mosaics, mCherry signaling existed in sections of adult ovaries from all strains (see Supplementary Fig. S1), while only one F1 strain using the *bactin2* promoter was retrieved from a total of 82 F0 adults (6/82), suggesting that proper dosage of transgene was required to generate a viable and fertile line. Distribution of mCherry-MBD driven by the *bactin2* promoter was further confirmed in both whole embryos and vital organs at adulthood (Fig. 1b,c). After several generations of outcrossing, the line was stabilized, and is referred to here as “zebraRDM”.

### Colocalization of mCherry-MBD with heterochromatin and modified histones

**Figure 2:**
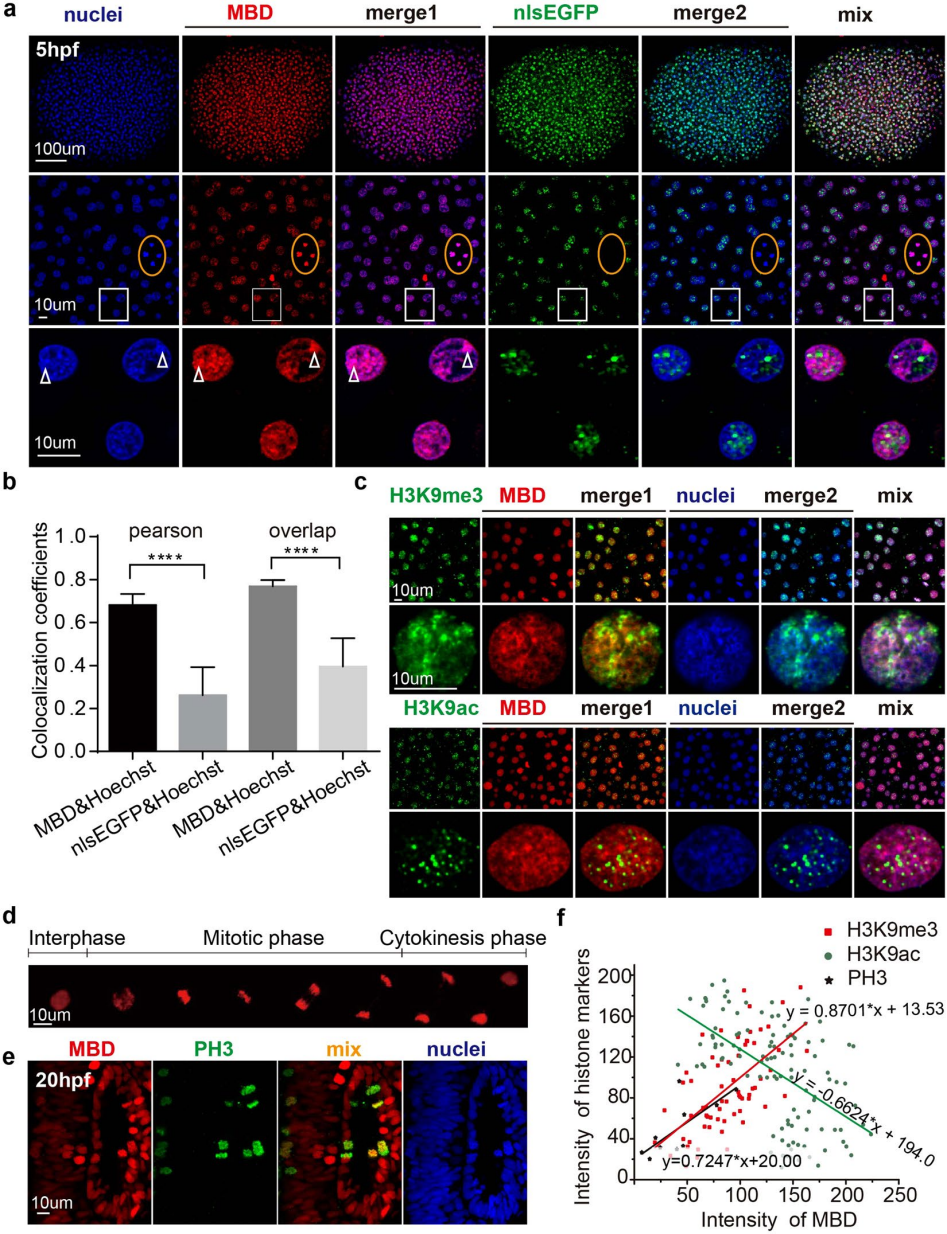
Colocalization of mCherry-MBD with heterochromatin and modified histones. (**a**) The correlation between Hoechst staining and the distribution patterns of the probes in 5 hpf embryos. Higher magnification images are arranged below. The orange boxes denote cells in mitotic metaphase; note that the nlsEGFP signal has disappeared due to nuclear envelope breakdown. White boxes below show magnified images. White arrowheads label puncta in Hoechst-stained nuclei. (**b**) Columns represent the overlap and Pearson coefficients for colocalization between Hoechst and the probes. P values < 0.0001, N = 27. (**c**) The colocalization pattern of mCherry-MBD and H3K9me3/ac. Lower rows showed subcelluar structure in one single cell nuclei. (**d**) Time lapse imaging of mCherry-MBD dynamics during the cell cycle. (**e**) The distribution of mCherry-MBD in phospho-Histone H3-positive metaphase cells. (**f**) The linear regression analysis between the intensity of MBD and that of modified histones. The plots represent cells or ROIs (region of interest) selected by image j (H3K9me3, N = 66 ROIs; H3K9ac, N = 112 ROIs; PH3, N = 10 cells). The intensity value ranged from 0 to 255. P value < 0.0001.

Heterochromatin is a highly compacted form of DNA enriched in epigenetic marks such as DNA and H3K9 methylation, which indicate a transcriptionally silenced region^10^. Studies have demonstrated that MBD1 is an important methyl-binding protein in heterochromatin formation, through interactions with methylated histones and chromatin remodeling proteins^23, 26^. We examined colocalization of the mCherry-MBD probe with heterochromatin in zebrafish embryos at 5 hours post fertilization (hpf). The mCherry-MBD probe accumulated gradually during cell proliferation, and its fluorescence could be easily detected by microscope as early as 4-5 hpf. As shown in Fig. 2a, both probes were expressed ubiquitously under control of the *bactin2* promoter in a global view. The nlsEGFP probe punctate dots throughout the nuclei and diffused away during mitosis (Fig. 2a, orange boxes). In comparison, mCherry-MBD highly colocalized with the puncta of Hoechst-stained nuclei, which marks heterochromatic regions. Quantitative colocalization analysis using randomly selected cells confirmed that mCherry-MBD highly colocalized with heterochromatin, while nlsEGFP did not (overlap of 0.78 + 0.01 and 0.44 + 0.02, respectively), and the intensity of mCherry-MBD was more correlated with the Hoechst fluorescence intensity than nlsEGFP (Pearson coefficients of r = 0.67 + 0.01 and r = 0.26 + 0.02 respectively; Fig. 2b). At 24 hpf and 48 hpf, both probes remained ubiquitously expressed, and mCherry-MBD intensity was also positively correlated with Hoechst fluorescence intensity in a general view (see Supplementary Fig. S2)^27^.

Compared with mammals, zebrafish had less apparent chromatin condensation within nuclei because of the lack of HP1 (heterochromatin protein1)^28^. As reported previously, the involvement of DNA methylation in heterochromatin formation was partly mediated by histone modifications^29–31^. To further study the relationship between the probe mCherry-MBD and heterochromatin structures, we conducted immunostaining on zebraRDM embryos using several representative histone markers, for example, the trimethylation of H3K4 and acylation of H3K9 well known as active promoters’ markers, and H3K9me2/3 or H3K27me3 representing the silent genes. From the microscopic view, the intensity of mCherry-MBD was significantly correlated to that of the constitutive heterochromatin marker H3K9me3 (Pearson r = 0.6249, N = 66), and negatively related to that of the active chromatin marker H3K4ac (Pearson r = −0.5403; Fig. 2c,f) in 5 hpf embryos. In addition, from the general view, the distribution pattern of mCherry-MBD was similar to that of the modified chromatin H3K27me3 (Pearson r = 0.8963, N = 30), and opposite to that of H3K4me3 (Pearson r = −0.4288, N = 26) (see Supplementary Fig. S3). It has also been reported that heterochromatin changes dynamically during mitosis, and methylated CpGs are propagated in nascent strands^32, 33^. In time-elapse imaging of a single zebraRDM cell at 6 hpf, we observed a transient increase in the fluorescence level at metaphase and anaphase (Fig. 2d). The observation was further verified by a positive correlation of mCherry-MBD intensity with phospho-Histone H3 serine 10 (PH3) staining (r = 0.74), which was used to label cells in mitosis (Fig. 2e,f).

### Comparison of mCherry-MBD and 5 mC distribution patterns

**Figure 3:**
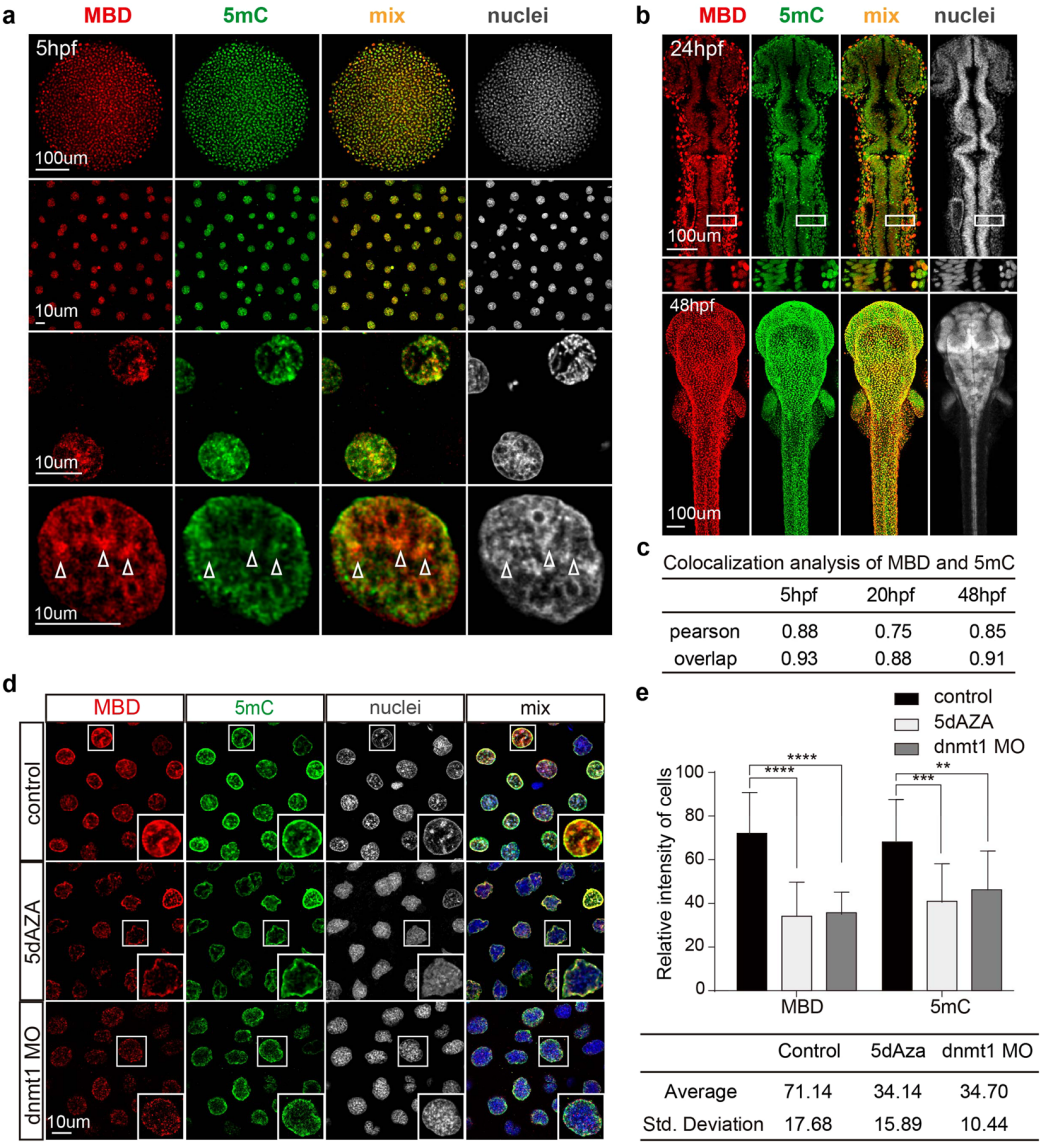
Comparison of the mCherry-MBD and 5 mC distribution patterns. (**a**) The distribution patterns of mCherry-MBD and anti-5 mC staining at 5 hpf. Higher magnification images are arranged below. White arrowheads label compacted puncta of chromatin. (**b**) The distribution patterns of mCherry-MBD and 5 mC at 24 hpf and 48 hpf. White boxes below are magnified 24 hpf images. (**c**) Colocalization analysis between mCherry-MBD, 5 mC, and heterochromatin (N = 12). (**d**) Comparison of nuclei from control embryos, and embryos treated by 5-dAZA or injected with dnmt1 MO. White boxes at right bottom are magnifications of each enclosed cell. (**e**) Fluorescence intensity of cells in each group (N = 16). The intensity value ranged from 0 to 255. P < 0.05. The intensity spectrums represented by the standard deviation are listed below.

Quantification of 5 mC is considered the gold standard for measuring DNA methylation status^34^. In MethylRO mouse ESCs, Yamagata *et al*. verified mCherry-MBD by comparing 5 mC MeDIP-seq data with RFP-mediated MeDIP-seq profiles^15, 20^. We performed whole embryo 5 mC staining, and visually analyzed the distributions of mCherry-MBD and 5 mC. In Fig. 3a, the distribution pattern of mCherry-MBD in subcellular structures was highly consistent with that of 5 mC at 5 hpf (overlap 0.93) and that of Hoechst stained heterochromatin puncta as shown above (overlap 0.92) (Figs 3c,2a). Images pictured by super-resolution microscopy in Fig. 3a captured more details within the nuclei. In addition, mCherry-MBD intensity displayed significant positive correlation with that of 5 mC (r = 0.88). Similar distribution patterns were maintained after the blastocyst stage, as shown in Fig. 3b,c (overlap 0.88 at 20 hpf, 0.91 at 48 hpf), and their intensities remained positively correlated at later embryonic stages (r = 0.75 at 20 hpf, r = 0.85 at 48 hpf).

The nucleotide analog 5-aza-2-deoxycytidine (5dAZA) is routinely used to interfere with DNA methylation, by incorporation into DNA or by inhibiting the activity of DNA methyltransferases (DNMTs), including DNMT1, DNMT3a and DNMT3b^35^. Among the DNMTs, DNMT1 plays an essential role in DNA methylation maintenance, while DNMT3a and DNMT3b affect *de novo* methylation. In our studies, both 5dAza and *dnmt1* morpholino (MO) were injected into the embryos at the single cell stage to down-regulate DNA methylation, after which the average intensities of mCherry-MBD and immunostained 5 mC significantly decreased (Fig. 3d). In addition, the standard deviation of the intensity values, which reflects the dynamic range of chromatin fluorescence, also decreased in the treated groups (Fig. 3e). The lower dynamic range suggests that heterochromatin formation was also inhibited, due to deficient methylation of CpG islands in 5dAza- or *dnmt1* MO-treated embryos. The average intensity values in the 5dAZA-treated group were comparatively lower than in the *dnmt1* morphants (Fig. 3e), suggesting that dnmt3a and dnmt3b, which are reported to be highly active in early embryos, may partially counter the effects of the *dnmt1* MO through *de novo* whole genome DNA methylation^36^.

### Differential methylated cells and gene loci recognized by the probe mCherry-MBD

**Figure 4:**
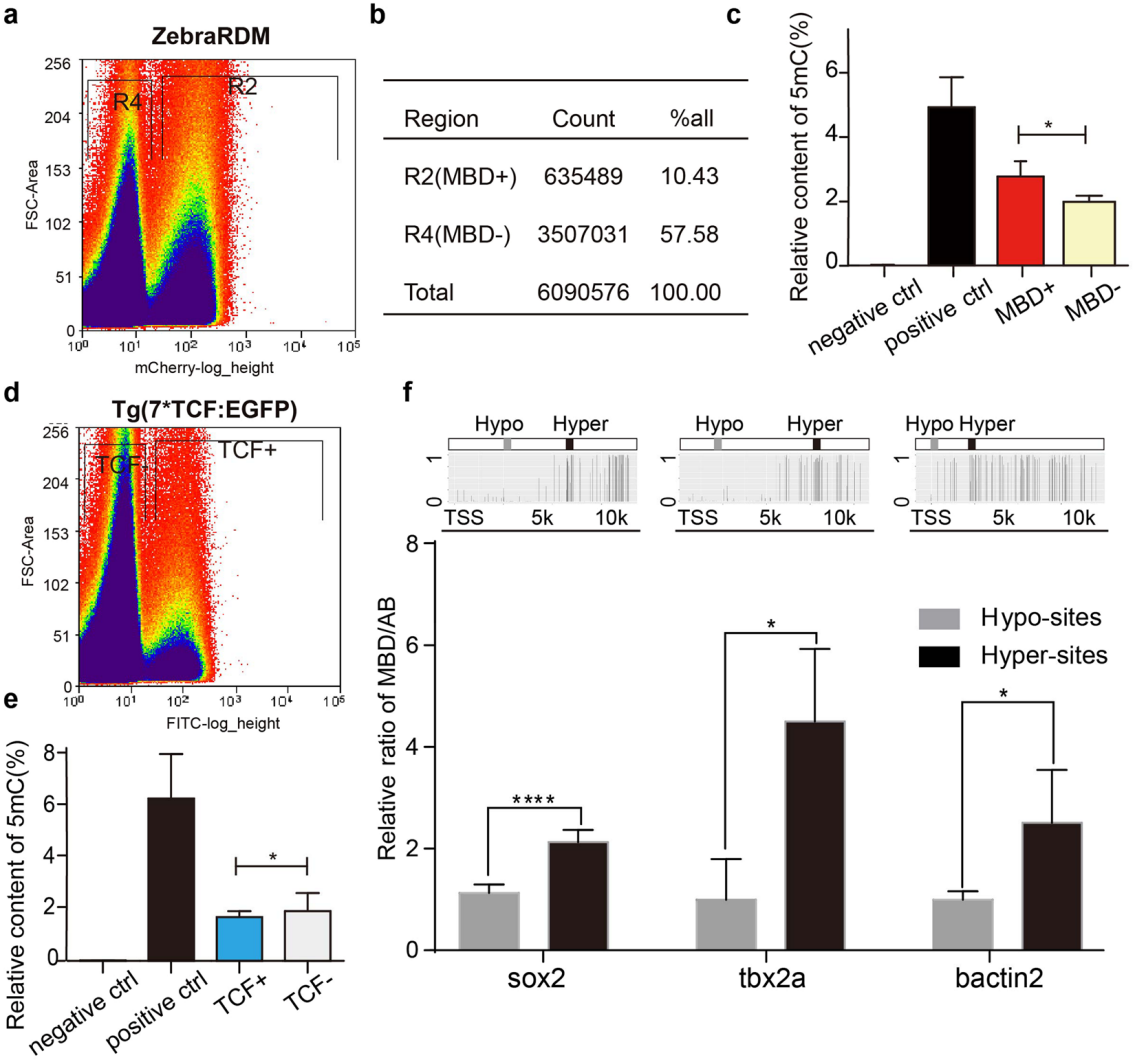
Differential methylated cells and gene loci recognized by the probe mCherry-MBD. (**a**) The mCherry fluorescence dot plot of cells in zebraRDM. R2, MBD+ group; R4, MBD− group. (**b**) Cell numbers and proportion of sorted cells. (**c**) Columns represent the percentage of 5 mC in DNA isolated from the MBD+ and MBD− groups (P = 0.026). (**d**) The GFP fluorescence dot plot of Wnt-responsive cells. (**e**) Columns represent the percentage of 5 mC in DNA isolated from TCF+ and TCF− groups (P = 0.0489). (**f**) The Chip-qPCR results of three genes′ hypo-/hyper-methylated sites. The images above are schematic pictures of three genes’ methylation status and their hypo-/hyper-methylated sites’ position. Columns represent the content of specific sites’ DNA fragment, normalized to the AB control. TSS, transcription start site; Hypo-sites, hypo-methylated sites; Hyper-sites, hyper-methylated sites.

The level of whole genome DNA methylation is often correlated to the differentiation status and stemness of certain cell lineages^37, 38^. We hypothesized that cell lineages with different levels of DNA methylation could be visually classified based on fluorescence intensity in zebraRDM. Firstly, we performed fluorescence-activated cell sorting (FACS) to sort cells from 72 hpf embryos into groups with strong (MBD+, R2) and weak (MBD−, R4) red fluorescence (Fig. 4a). After adjusting for interference from autofluorescence, 10.43% of the overall cell population was defined as MBD+ (Fig. 4b). Genomic DNA was isolated from both groups to quantify their methylated cytosine. As shown in Fig. 4c, the genomic DNA methylation level, represented by the percentage of 5 mC, was significantly higher in the MBD+ group than in the MBD− group (2.76 + 0.27 and 1.78 + 0.10, respectively; p = 0.026). Hence, by using FACS or observing probe intensity, we could measure the global DNA methylation status in specific cell lineages. We next investigated the methylation status in various stem cell niches during development. The canonical Wnt/beta-catenin signaling pathway is broadly activated in progenitor cells, and here we sorted Wnt-responsive cells from 3 dpf embryos of a Wnt reporter zebrafish line, *Tg(7xTCFX.laSiam:EGFP)* ^*ia4*^ ^39, 40^. The Wnt-responsive cells (R2, TCF+ group), which represented 2.92% of all somatic cells (Fig. 4d), showed a lower global DNA methylation level than TCF− group (2.14 + 0.12 and 2.43 + 0.39, respectively; P = 0.0489; Fig. 4e).

To better validate the authenticity of this reporter, we further performed mCherry-mediated Chip test, and the pull-down DNA was used to detect candidate gene sites’ content by qPCR. We analyzed a published whole-genome bisulfite sequencing data obtained from zebrafish embryos, submitted under GEO accession id GSE74789^41^. Three genes-sox2, tbx2a, bactin2 were selected for their high expression in early embryonic cells. Results showed that the DNA methylation status in specific loci ranged from TSS (transcriptional start site) to 10 k upstream did not change much from 4 hpf to 24 hpf (see Supplementary Figure S4a). The Chip-test was performed on 24 hpf zebraRDM transgenic embryos using mCherry antibody, and the wild type (AB) embryos as a negative control. According to the methylation status, Candidate qPCR sites were classified to hypo-methylated sites (Hypo-sites) and hyper-methylated sites (Hyper-sites). Specific primers were designed and could be accessed in the Supplementary Figure S5. For each of gene site, four groups including input, MBD, AB, IgG were analyzed (see Supplementary Figure S4b). The results showed that hyper-methylated DNA fragments were significantly enriched in the mCherry-mediated Chip products, while the hypo-methylated fragments were not (Fig. 4f). The relative content ratio of hyper- and hypo-sites were: *sox2* (2.133 + 0.1370 and 1.129 + 0.06727 N = 6), *tbx2a* (4.506 + 0.8217 and 1.000 + 0.3240, N = 3), *bactin2* (2.512 + 0.4225 and 1.000 + 0.08055 N = 6). The results honestly confirmed that hyper-methylated DNA fragments were in higher enrichment in the mCherry pull-down DNA. These biochemical evidences can support that the mCherry-MBD specifically bound to the methylated DNA.

### Differential DNA methylation levels in the developing heart

**Figure 5:**
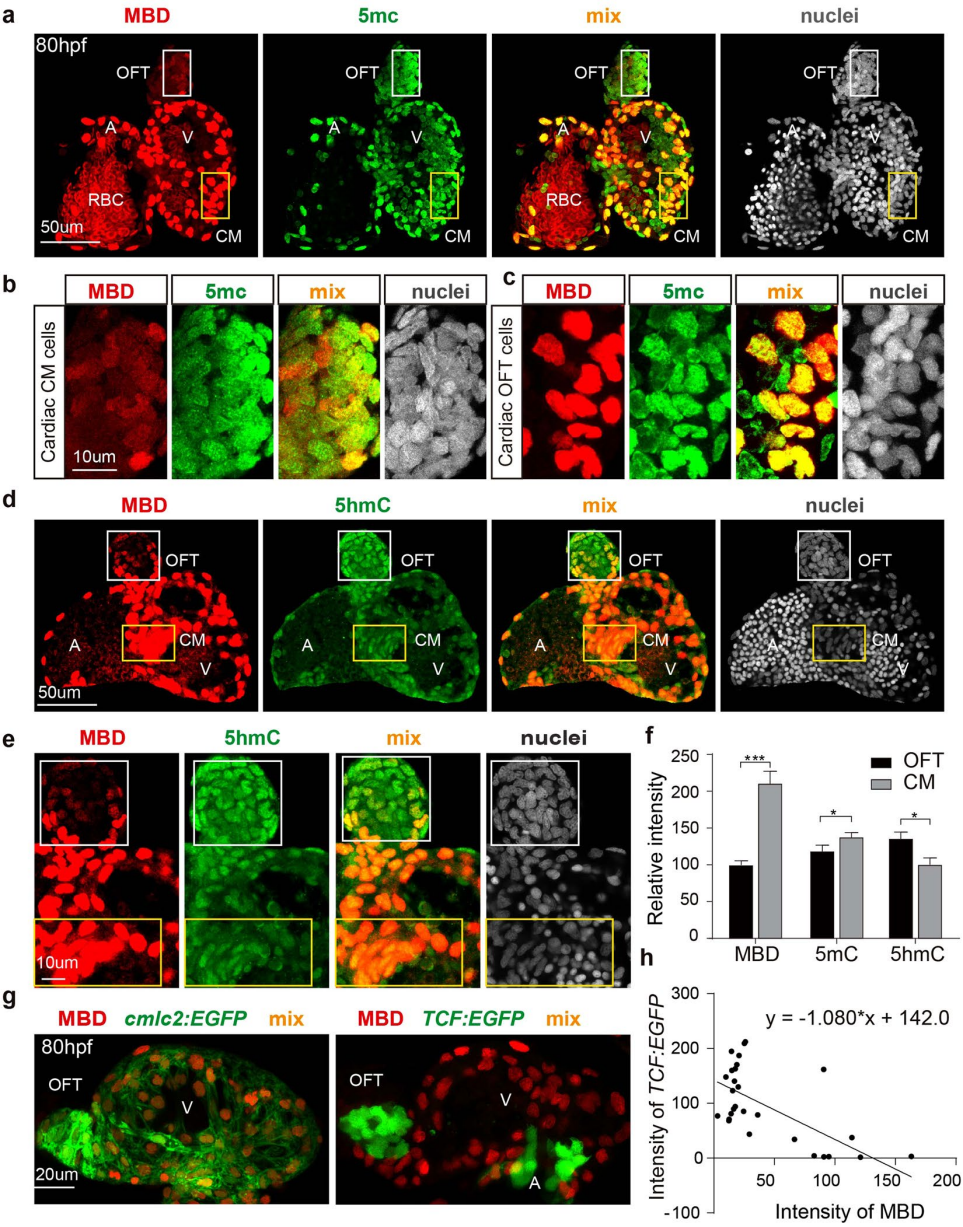
DNA methylation and hydroxymethylation patterns in the developing heart. (**a**) The distribution patterns for mCherry-MBD and 5 mC in the 80 hpf heart. (**b**,**c**) Magnified images of cardiac outflow tract cells (white boxes) and cardiac muscle cells (orange boxes) in A. A, atrium; V, ventricle; OFT, out flow tract; CM, cardiac muscle. (**d**) Comparison of mCherry-MBD and 5 hmC in the 80 hpf heart. (**e**) Magnified images of cardiac outflow tract cells (white boxes) and cardiac muscle cells (orange boxes) in D. (**f**) Fluorescence intensities of each group in the OFT and CM (p = 0.0105; N = 3) (**g**) The positional relationship of Wnt-responsive cells and mCherry-MBD expressing cells in 80 hpf heart. Left, cardiac muscle cells marked by *cmlc2:EGFP*; right, Wnt-responsive cells marked by *TCF:EG*FP. (**h**) Linear regression of TCF and MBD signaling intensity (P value < 0.0001, N = 30).

Given that stem and progenitor cells are generally hypo-methylated, the zebraRDM model was used to help distinguish different cell lineages within a specific organ^42, 43^. As a vital organ developed at early stages, with relatively simple cellular composition, the heart was selected to visualize differences in methylation status. The distribution of mCherry-MBD followed a similar pattern to that of immunostained 5 mC in distinct cell groups, including cardiac outflow tract cells and muscle cells (Fig. 5a–c). Both the probe and 5 mC maintained higher concentrations in cardiac muscle (CM) cells and were only weakly detected in outflow tract (OFT) regions, with the intensity of mCherry-MBD (CM 209.4 + 10.44 and OFT 98.56 + 3.38) more sensitive to methylation variance than that of 5 mC immunostaining (CM 136.6 + 3.94 and OFT 117.7 + 5.02; Fig. 5d,e). In contrast with mCherry-MBD and 5 mC, 5-hydroxymethylcytosine (5 hmC) was more enriched in cardiac outflow tract cells than in muscle cells (OFT134.5 + 5.56 and CM 99.06 + 5.49), confirming that DNA methylation undergoes dynamic changes during cell differentiation^44, 45^. We next crossed zebraRDM with *Tg(7xTCFX.laSiam:EGFP)* ^*ia4*^ to examine the embryonic heart at 80 hpf, and observed that the TCF+ cells, representing the stem cell niche in the heart, mostly aggregated in the outflow tract and atrioventricular junction^46^. We found that TCF+ cells and MBD+ cells had mutually exclusive distribution patterns, and regression analysis indicated that the intensity of Wnt-responsive cells was negatively correlated with that of mCherry-MBD (r = −0.6583; Fig. 5h), confirming the biochemical results above (Fig. 4e). All these results suggest that the stem cell niches of organs experience intricate modulation of DNA methylation during organogenesis.

## Discussion

Many studies have emphasized important roles for DNA methylation in almost all developmental and pathological processes^47^. However, understanding of the detailed mechanisms remains elusive, due to a lack of convenient approaches to directly investigate the dynamic process *in vivo*. As a widely accepted model for developmental genetics and human diseases, the zebrafish serves as a promising platform for epigenetic research^19^. Therefore, in this work, we attempted to generate several lines of transgenic zebrafish ubiquitously expressing an mCherry-MBD probe. Surprisingly (but luckily), one stable strain using the *bactin2* promoter was retrieved from nearly a hundred F0 mosaics. Named zebraRDM, it was used to perform high resolution imaging of DNA methylation dynamics in embryonic development and cell lineage commitment.

Currently, mouse models are widely used in DNA methylation and stem cell research, but high-resolution visual investigation of methylation dynamics in live mice remains challenging at later stages^48^. In comparison, zebrafish have significant advantages, such as embryonic transparency and quick development, and our zebraRDM reporter provides an opportunity to better understand epigenetic changes during cell state transitions. The reporter allows visual assessment of the DNA methylation state with subcellular resolution, as well as FACS sorting based on mCherry fluorescence intensity, and our results suggested that cells with stronger mCherry fluorescence had higher methylation status. DNA methylation serves as good indicator of cell differentiation status, and it is commonly regarded that stem cells maintain a lower DNA methylation level^42, 43, 49^. Here, a transgenic canonical Wnt reporter was used to label embryonic progenitor cells at 72 hpf, and Wnt-responsive cells in the developing heart displayed weaker mCherry-MBD fluorescence intensity, indicating a lower DNA methylation level^39, 50^. In addition, correlations between mCherry-MBD, 5 mC, and 5 hmC intensities were precisely demonstrated in the cardiac muscle and cardiac outflow tract regions, suggesting that the zebraRDM reporter can be used to study DNA methylation dynamics in developing organs, with multiple potential applications.

The relationships between DNA methylation, heterochromatin, and certain histone modifications were traditionally investigated via biochemical approaches^23, 26^. The zebraRDM reporter provides an alternative approach, enabling the capture of high-resolution images of the distribution pattern of the mCherry-MBD probe and the structure of heterochromatin. Statistical analyses demonstrated that the probe highly colocalized with heterochromatin, and both probe distribution and heterochromatin formation can be inhibited using either a demethylating agent or *dnmt1* MO. Furthermore, DNA methylation is also correlated with certain histone modifications, and correspondingly, we observed the mCherry-MBD probe in a similar distribution pattern to the constitutive heterochromatin marker H3K9me3, opposing that of the active chromatin marker H3K9ac. A temporary DNA methylation increase is required for mitosis, and we also observed higher methylation status in mitotic cells through time-lapse imaging of blastulas^33^.

Unexpectedly, the MBD domain appears to be toxic for germ line or early embryonic development, and it took tremendous screening effort to produce stabilized and viable offspring. In the future, a conditional transgenic expression strategy using a tetracycline-regulated system or *hsp70l* promoters could be used to avoid this problem. Additionally, the reportedly ubiquitous *bactin2* promoter has tissue-specific variations in expression, making quantitative comparison of cells from different tissues quite difficult^51^. However, our studies suggest that this reporter is a powerful and convenient platform for investigating DNA methylation. With the application of super-resolution microscopy techniques, zebraRDM may prove extremely valuable in uncovering the molecular mechanisms behind dynamic changes in DNA methylation.

## Materials and Methods

### Zebrafish strains

Wild-type zebrafish (AB strain) were obtained from the Zebrafish International Resource Center (ZIRC, Oregon, USA). Zebrafish embryos, larvae, and adults were collected, staged and maintained as previously described^52^. In some cases, embryos and larvae were initially raised in water containing 0.003% 1-phenyl-2-thiourea (PTU, P3755; Sigma, USA) to prevent pigment formation. Published strains used in this study include *Tg(cmlc2:EGFP)* and *Tg(7xTCFX.laSiam:EGFP)* ^*ia446*^. All studies involving animal manipulations were approved by the Fudan University Shanghai Medical College Animal Care and Use Committee, and followed the National Institutes of Health guidelines for the care and use of animals.

### Plasmid constructs

Targeting vectors were constructed using the Tol2-based multisite gateway technique^53^. The pcDNA3.1 mCherry-nls-MBD1 vector was a generous gift from Professor Kazuo Yamagata at Osaka University (Japan), and entry clones, vectors, and the Multisite Gateway® Three-Fragment Vector Construction Kit (Catalog no. 12537-023) were purchased from Invitrogen (USA). We inserted *bactin2, hsp70* and *ef1a* promoters in the 5′ entry clones to ubiquitously drive the expression of the fused protein mCherry-nls-MBD1 in the middle entry clone, and at the same time, the expression of the 3′ IRES-nlsEGFP.

### Generation of *Tg(bactin2:mCherry-MBD-IRES-nlsEGFP)* Transgenic Lines

For the generation of zebraRDM, the constructed vectors (50 ng/egg) and transposase mRNA (100 ng/egg) were co-injected into single cell stage embryos. Embryos and larvae were examined using an Olympus SZX12 microscope (Olympus, Japan), with a GFP filter or RFP filter, and photographed using a DP70 digital camera (Olympus, Japan). Fluorescence of mCherry-MBD could be directly observed in living embryos, but the intensity of IRES-nlsEGFP was too weak for direct observation, requiring GFP immunostaining for signal amplification. Adult F0 transgenic zebrafish were crossed with wild-type zebrafish to obtain subsequent generations.

### 5dAza and *dnmt1* MO delivery

5dAza (Sigma, USA) was suspended in sterile water to a concentration of 100 μM and aliquoted to avoid multiple freeze/thaws. Aliquots were stored at −80 °C. Zebrafish embryos were injected with 3 L of suspended 5dAza into the yolk at the single cell stage. Dnmt1 MO (Gene Tools, USA) were dissolved in nuclease-free water at 0.125 mM, and 16 ng *dnmt1* MO was injected into each embryo at the single cell stage.

### Immunofluorescence

Zebrafish embryos at certain stages were dechorionated manually, and fixed by 4% paraformaldehyde for 1 day at 4 °C. Fixed embryos were washed with 0.1% PBST (0.1% Tween-20 in PBS) three times, then digested with 0.05% collagenase II (Gibco, USA). Penetrated embryos were then incubated in 2 M HCl for 30 min at room temperature (22–25 °C) followed by hydration for 5 min. Next, embryos were washed with 0.1% PBST three times for 5 min each, and incubated in blocking buffer (0.5% DMSO, 0.5% Triton X-100, 5% goat serum in PBS) for 1 h. After incubation, the embryos were incubated in blocking buffer containing primary antibodies overnight at 4 °C, followed by washing with 0.1% PBST two times for 5 min then four times for 30 min. Next, embryos were incubated with fluorescence-conjugated secondary antibodies overnight at 4 °C. After washing with 0.1% PBST six times, embryos were mounted in glycerol and observed by confocal microscopy (Leica TCS SP8, Germany). Confocal laser scanning was done on a Leica-4 channel system controlled by LAS AF Lite_2.6.0_7266 software. The high-resolution images were pictured and processed by (Leica TCS SP5, Germany). Image processing and intensity measurements were done using ImageJ software.

The following primary antibodies were used: mouse anti-5 mC (Abcam, UK; 1:500), rabbit anti-EGFP (Abcam, UK; 1:500), rabbit anti-H3K4me3 (Abcam, UK; 1:100), rabbit anti-H3K27me3 (Millipore, USA; 1:100), rabbit anti-H3K9me3 (Abcam, UK; 1:1000), rabbit anti-H3K9ac (Abcam, UK; 1:1000), rabbit anti-phospho-Histone H3 (Ser10) (Cell Signaling Technology, USA; 1:500). Goat serum and Secondary antibodies including Alexa Fluor 488-conjugated anti-mouse, Cyanine Cy™ 3 anti-mouse, Alexa Fluor 647-conjugated anti-mouse, Alexa Fluor 488-conjugated anti-rabbit, and Alexa Fluor 647-conjugated anti-rabbit secondary antibodies (Jackson ImmunoResearch, USA; all diluted 1:500 for use). Nuclei were stained with Hoechst 33342 (Invitrogen, USA; 1:500).

### Fluorescence-activated cell sorting

Zebrafish embryos at 72 hpf were digested in 0.25% trypsin EDTA solution (Thermo Fisher Scientific, USA) on an orbiter for 30 min at room temperature(22–25 °C), then filtered through 40 μm cell strainers (BD, USA) into 50 mL tubes. Collected cells were then centrifuged at 1,200 rpm for 5 min at 4 °C. The supernatant was removed and cells were washed once in PBS. Resuspended cells were filtered again and analyzed on a MoFlo XDP cell sorter (Beckman-Coulter, USA). MCherry was excited with a 100 mW 561 nm argon laser and detected in FL9 (625+/− 26 nm). Collected cells were sorted into MBD1- (R4 region) and MBD1 + (R2 region) groups according to mCherry intensity. Wnt-responsive cells were detected by EGFP with a 100 mW 488 nm argon laser, and detected in a 525+/− 25 nm bypass filter.

### Methylated DNA quantification

Whole genome DNA was isolated from sorted cells using the QIAamp DNA Mini kit (Qiagen, USA). DNA concentration and purity were determined by comparing the ratio of optical density measurements at 260 and 280 nm. Global DNA methylation status was detected using the MethylFlash Methylated DNA Quantification Kit (Colorimetric) (Epigentek, USA). In this assay, 100 ng of genomic DNA from each group was added into strip wells treated to have high DNA affinity. The methylated fraction of DNA was detected using capture and detection antibodies and then quantified colorimetrically by reading the absorbance in a Enspire microplate spectrophotometer (PerkinElmer, USA) at 450 nm.

### Chip-qPCR

Chip test was performed using Epiquik Tissue Chip Kit (Epigentek, USA). Zebrafish embryos at 24 hpf were collected and dechorionated manually, followed by *in vivo* cross-link process with 1 ml 1% formaldehyde solution incubating for 15–20 min on a rocking platform. After washing by 1 ml 125 mM Glycine solution for 5 min, the embryos were homogenized by adding 1 ml Homogenizing Buffer. The homogenized mixture was centrifuged to remove supernatant, and the disaggregated tissue pellet was resuspended by 500 ul Lysis buffer. After incubation for 15 min on ice, the mixture was sheared using the Bioruptor® ultrasonicator (Diagenode, Belgium). The sonication factor is: 30 s work, 30 s intervals, 5cycles, rest for 2 min; repeat the work. The length of sheared DNA should be between 200–1000 bp. Sheared DNA could be used to do the protein-DNA immunoprecipitation and DNA reversal. These progresses were performed according to the Epiquik Tissue Chip Kit manual book.

The primers for qPCR was designed in primer premier 5.0 (see Supplementary Figure S4).

### Quantification and statistical analysis

Colocalization analyses was performed using the plugin JACoP of ImageJ software^27^. Statistical differences between groups were determined using t test through GraphPad Prism 6 (GraphPad Software), and P < 0.05 were considered significant. Multiple curve fitting was performed in the software OriginPro 9.0.0.

## Electronic supplementary material

Supplementary files

## References

[1] Gohlke J (2013). DNA methylation mediated control of gene expression is critical for development of crown gall tumors. PLoS genetics.

[2] Chen RZ, Pettersson U, Beard C, Jackson-Grusby L, Jaenisch R (1998). DNA hypomethylation leads to elevated mutation rates. Nature.

[3] Maunakea AK (2010). Conserved role of intragenic DNA methylation in regulating alternative promoters. Nature.

[4] Costello JF (2000). Aberrant CpG-island methylation has non-random and tumour-type-specific patterns. Nature genetics.

[5] Sansom OJ, Maddison K, Clarke AR (2007). Mechanisms of disease: methyl-binding domain proteins as potential therapeutic targets in cancer. Nature clinical practice. Oncology.

[6] Allan AM (2008). The loss of methyl-CpG binding protein 1 leads to autism-like behavioral deficits. Hum Mol Genet.

[7] Baubec T, Ivanek R, Lienert F, Schubeler D (2013). Methylation-dependent and -independent genomic targeting principles of the MBD protein family. Cell.

[8] Bird A (2002). DNA methylation patterns and epigenetic memory. Genes Dev.

[9] Roloff TC, Ropers HH, Nuber UA (2003). Comparative study of methyl-CpG-binding domain proteins. BMC genomics.

[10] Du Q, Luu PL, Stirzaker C, Clark SJ (2015). Methyl-CpG-binding domain proteins: readers of the epigenome. Epigenomics.

[11] Ohki I (2001). Solution structure of the methyl-CpG binding domain of human MBD1 in complex with methylated DNA. Cell.

[12] Frommer M (1992). A genomic sequencing protocol that yields a positive display of 5-methylcytosine residues in individual DNA strands. Proc Natl Acad Sci USA.

[13] Jorgensen HF, Adie K, Chaubert P, Bird AP (2006). Engineering a high-affinity methyl-CpG-binding protein. Nucleic Acids Res.

[14] Clark C (2012). A comparison of the whole genome approach of MeDIP-seq to the targeted approach of the Infinium HumanMethylation450 BeadChip((R)) for methylome profiling. PLoS One.

[15] Rauch TA, Pfeifer GP (2010). DNA methylation profiling using the methylated-CpG island recovery assay (MIRA). Methods.

[16] Li N (2010). Whole genome DNA methylation analysis based on high throughput sequencing technology. Methods.

[17] Ueda J (2014). Heterochromatin dynamics during the differentiation process revealed by the DNA methylation reporter mouse, MethylRO. Stem Cell Reports.

[18] Stelzer Y, Shivalila CS, Soldner F, Markoulaki S, Jaenisch R (2015). Tracing dynamic changes of DNA methylation at single-cell resolution. Cell.

[19] Kamstra JH, Alestrom P, Kooter JM, Legler J (2015). Zebrafish as a model to study the role of DNA methylation in environmental toxicology. Environ Sci Pollut Res Int.

[20] Yamagata K (2010). DNA methylation profiling using live-cell imaging. Methods.

[21] Sasai N, Defossez PA (2009). Many paths to one goal? The proteins that recognize methylated DNA in eukaryotes. The International journal of developmental biology.

[22] Jorgensen HF, Ben-Porath I, Bird AP (2004). Mbd1 is recruited to both methylated and nonmethylated CpGs via distinct DNA binding domains. Molecular and cellular biology.

[23] Hameed UF (2014). Transcriptional repressor domain of MBD1 is intrinsically disordered and interacts with its binding partners in a selective manner. Sci Rep.

[24] Kwan KM (2007). The Tol2kit: a multisite gateway-based construction kit for Tol2 transposon transgenesis constructs. Dev Dyn.

[25] Higashijima S, Okamoto H, Ueno N, Hotta Y, Eguchi G (1997). High-frequency generation of transgenic zebrafish which reliably express GFP in whole muscles or the whole body by using promoters of zebrafish origin. Dev Biol.

[26] Grewal SI, Jia S (2007). Heterochromatin revisited. Nat Rev Genet.

[27] Bolte S, Cordelieres FP (2006). A guided tour into subcellular colocalization analysis in light microscopy. Journal of microscopy.

[28] Gilbert N (2003). Formation of facultative heterochromatin in the absence of HP1. The EMBO journal.

[29] Liu Y (2007). RNAi-dependent H3K27 methylation is required for heterochromatin formation and DNA elimination in Tetrahymena. Genes Dev.

[30] Deb M (2014). Chromatin dynamics: H3K4 methylation and H3 variant replacement during development and in cancer. Cellular and molecular life sciences: CMLS.

[31] Karmodiya K, Krebs AR, Oulad-Abdelghani M, Kimura H, Tora L (2012). H3K9 and H3K14 acetylation co-occur at many gene regulatory elements, while H3K14ac marks a subset of inactive inducible promoters in mouse embryonic stem cells. BMC genomics.

[32] Smith ZD, Meissner A (2013). DNA methylation: roles in mammalian development. Nat Rev Genet.

[33] Kishikawa, S., Murata, T., Ugai, H., Yamazaki, T. & Yokoyama, K. K. Control elements of Dnmt1 gene are regulated in cell-cycle dependent manner. *Nucleic acids research. Supplement (2001)* 307–308 (2003). 10.1093/nass/3.1.307 14510503

[34] Fraga, M. F. & Esteller, M. DNA methylation: a profile of methods and applications. *BioTechniques* **33**, 632, 634, 636–649 (2002). 10.2144/02333rv01 12238773

[35] Potok ME, Nix DA, Parnell TJ, Cairns BR (2013). Reprogramming the maternal zebrafish genome after fertilization to match the paternal methylation pattern. Cell.

[36] Okano M, Bell DW, Haber DA, Li E (1999). DNA methyltransferases Dnmt3a and Dnmt3b are essential for de novo methylation and mammalian development. Cell.

[37] Smith ZD (2014). DNA methylation dynamics of the human preimplantation embryo. Nature.

[38] Jiang L (2013). Sperm, but not oocyte, DNA methylome is inherited by zebrafish early embryos. Cell.

[39] Moro E (2012). *In vivo* Wnt signaling tracing through a transgenic biosensor fish reveals novel activity domains. Dev Biol.

[40] Goessling W (2009). Genetic interaction of PGE2 and Wnt signaling regulates developmental specification of stem cells and regeneration. Cell.

[41] Kaaij LJ (2016). Enhancers reside in a unique epigenetic environment during early zebrafish development. Genome biology.

[42] Ziller MJ (2011). Genomic distribution and inter-sample variation of non-CpG methylation across human cell types. PLoS genetics.

[43] Ji H (2010). Comprehensive methylome map of lineage commitment from haematopoietic progenitors. Nature.

[44] Chan MM, Smith ZD, Egli D, Regev A, Meissner A (2012). Mouse ooplasm confers context-specific reprogramming capacity. Nature genetics.

[45] Calvanese V (2012). A promoter DNA demethylation landscape of human hematopoietic differentiation. Nucleic Acids Res.

[46] Zhang RR, Gui YH, Wang X (2015). [Role of the canonical Wnt signaling pathway in heart valve development]. Zhongguo dang dai er ke za zhi = Chinese journal of contemporary pediatrics.

[47] Barua S, Junaid MA (2015). Lifestyle, pregnancy and epigenetic effects. Epigenomics.

[48] Lee DS (2014). An epigenomic roadmap to induced pluripotency reveals DNA methylation as a reprogramming modulator. Nat Commun.

[49] Tucker KL (1996). Germ-line passage is required for establishment of methylation and expression patterns of imprinted but not of nonimprinted genes. Genes Dev.

[50] Kwon C (2009). A regulatory pathway involving Notch1/beta-catenin/Isl1 determines cardiac progenitor cell fate. Nat Cell Biol.

[51] Ferguson-Smith AC (2011). Genomic imprinting: the emergence of an epigenetic paradigm. Nat Rev Genet.

[52] Westerfield, M. The zebrafish book: a guide for the laboratory use of zebrafish (Brachydanio rerio) (1994).

[53] Katzen F (2007). Gateway((R)) recombinational cloning: a biological operating system. Expert opinion on drug discovery.

