# Supplementary material for: High Resolution Imaging of DNA Methylation Dynamics using a Zebrafish Reporter
Source: Sci Rep. 2017 Jul 14;7:5430. doi: 10.1038/s41598-017-05648-8 (PMC5511286; doi:10.1038/s41598-017-05648-8)
Supplement: Supplementary file 1 — Supplementary files [file 41598_2017_5648_MOESM1_ESM.pdf]

High Resolution Imaging of DNA Methylation Dynamics using a Zebrafish Reporter

Ranran Zhang[1], Lian Liu [1], Yuxiao Yao[2], Fei Fei[2], Feng Wang[1], Qian Yang[1], Yonghao Gui[1*], Xu Wang[2*]

Supplementary Fig.S1 The distribution of the probe mCherry-MBD in F0 mosaic's ovaries

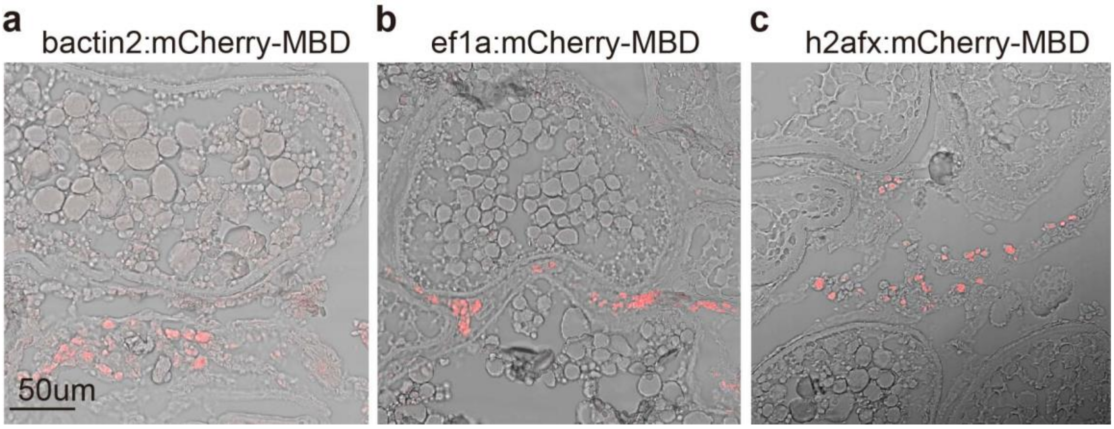

Fig.S1 (a-c) The distribution of the probe mCherry-MBD in the ovaries' section of adult *Tg*(*bactin2:mCherry-MBD-IRES-nlsEGFP*), *Tg*(*ef1a:mCherry-MBD-IRES-nlsEGFP*), and *Tg*(*h2afx:mCherry-MBD-IRES-nlsEGFP*) respectively.

Supplementary Fig.S2 The distribution pattern of the probes mCherry-MBD and nlsEGFP at 20hpf and 48hpf

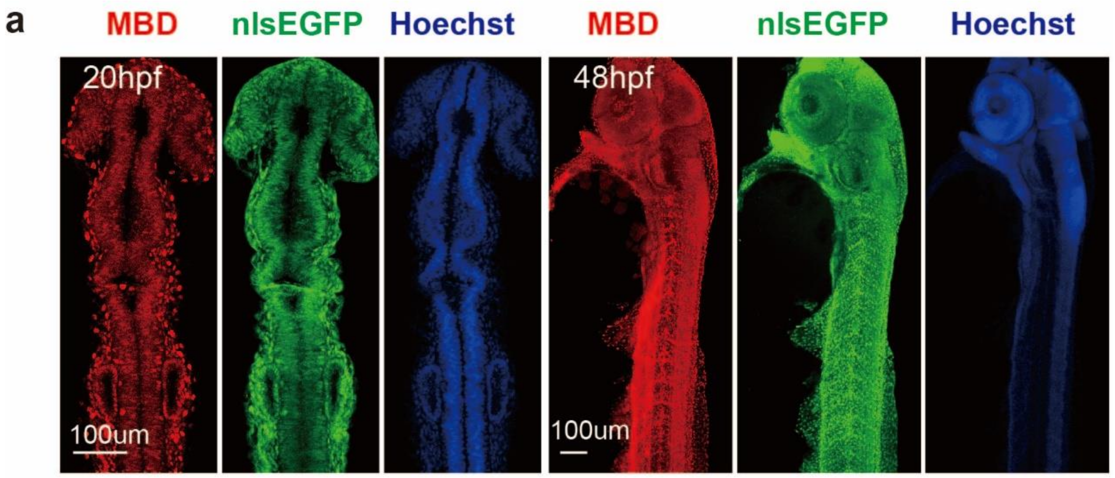

b

Colocalization coefficients at 20hpf and 48hpf

	20hpf		48hpf	
	pearson	overlap	pearson	overlap
MBD & Hoechst	0.749	0.826	0.601	0.734
EGFP & Hoechst	0.692	0.790	0.611	0.741

Fig.S2 (a) The distribution pattern of the probe mCherry-MBD and the probe nlsEGFP in embryos at 20hpf and 48hpf. **(b)** The pearson's correlation and overlap coefficient of chromosome and mCherry-MBD.

Supplementary Fig.S3 The correlation of tri-methylated H3K4/H3K27 with mCherry-MBD at 20hpf

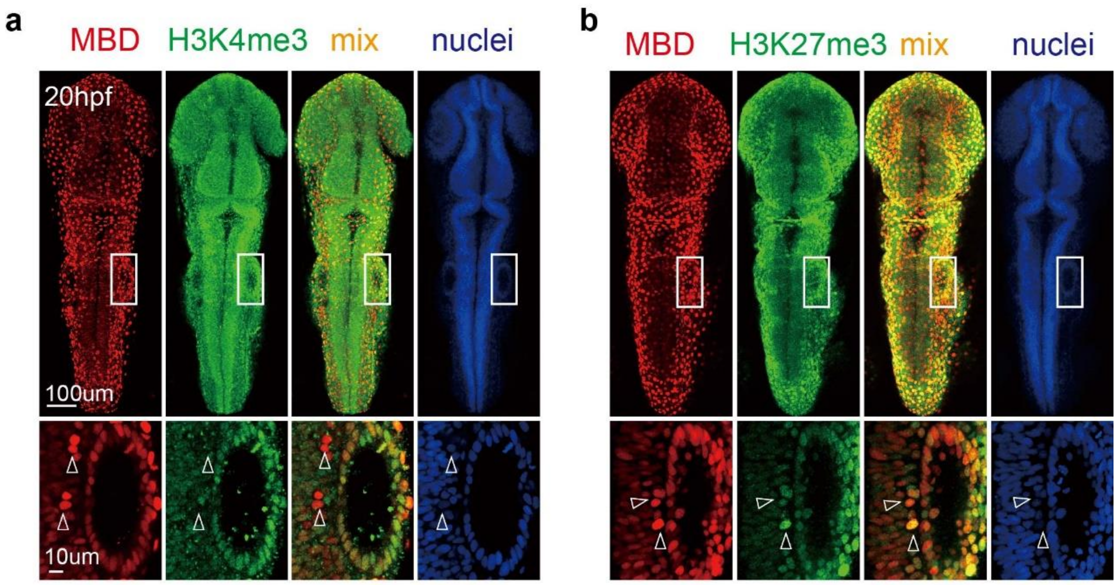

c

Correlation of histone modification markers with MBD

	Pearson r	95% confidence interval	p-value
MBD & H3K4me3	**-0.4162**	-0.6920 to -0.0343	0.0288
MBD & H3K27me3	**0.8963**	0.7917 to 0.9499	<0.0001

Fig.S3 (a) The correlation between mCherry-MBD and H3K4me3 at 20hpf. The images in white boxes are magnified below. White arrowheads denote overlapping cells. **(b)** The correlation between mCherry-MBD and H3K27me3 at 20hpf. **(c)** The pearson's correlation and overlap coefficient of trimethylated H3K4/H3K27 and mCherry-MBD in general view.

Supplementary Fig.S4 Specific genes' methylation status and the enrichment in mCherry-Chip DNA.

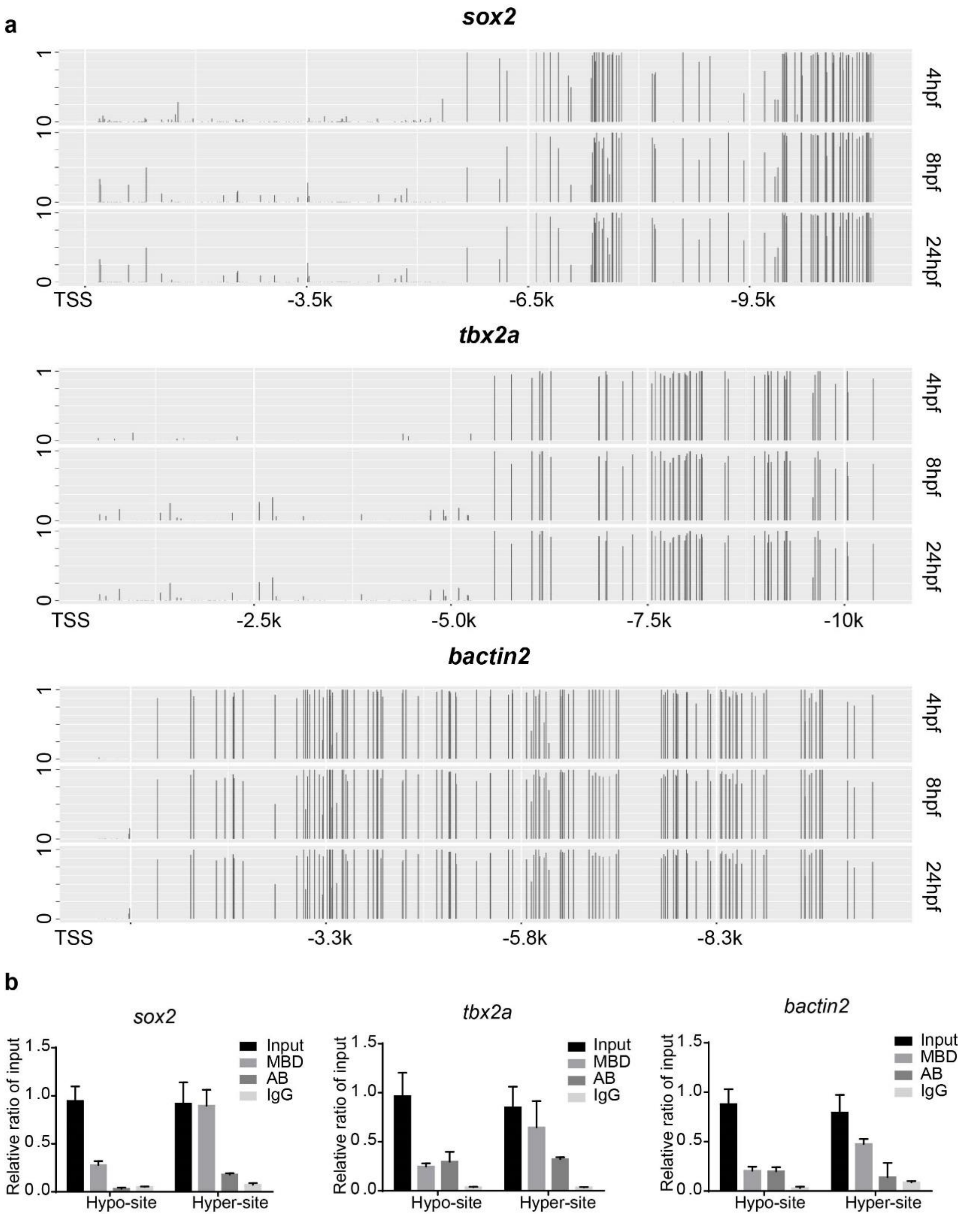

Fig.S4 (a) DNA methylation status in the upstream of TSS from genes *sox2, tbx2a, bactin2* at 4hpf, 8hpf and 24hpf. TSS, transcriptional start site. **(b)** The enrichment of hypo-/hyper-methylated sites from genes *sox2, tbx2a, bactin2* through mCherry-mediated Chip-qPCR. Columns represent the enrichment level of specific sites in different group. The columns were normalized to their input group. Hypo-site, hypomethylated site; Hyper-site, hypermethylated site.

Supplementary Table 1 Primers for Chip-qPCR

primer name	primer sequence
sox2 hypo F	ACCCCCACCAAACATAATGA
sox2 hypo R	AAAAAGCCAGCCCATTACCT
sox2 hyper F	GGCCTGGATTGCTGATGTAT
sox2 hyper R	CATTGAGGGTTTGCAGGAGT
tbx2 hypo F	AGCCATTAATTCCACATTGC
tbx2 hypo R	ACCGACAGGCAGAGGAAAAT
tbx2 hyper F	CCCTGAATGATCAGCTCCAT
tbx2 hyper R	CAGTTTCGACCTTGCAGTGA
bactin2 hypo F	TGGAATTTGCCAAAAACAAA
bactin2 hypo R	TGTGCTTGAGCAGCAGTGAT
bactin2 hyper F	GCTTCCCCTTCTTTCACTCTC
bactin2 hyper R	CATACCCGGCTCTAGCCATA
